# HGA-Induced Oxidative Stress Impairs Autophagy via Lysosomal Dysfunction in Alkaptonuria

**DOI:** 10.3390/antiox15070823

**Published:** 2026-06-30

**Authors:** Pierfrancesco Mastroeni, Alfonso Trezza, Anna Visibelli, Michela Geminiani, Annalisa Santucci

**Affiliations:** 1 ONE-HEALTH Lab, Department of Biotechnology, Chemistry and Pharmacy, University of Siena, Via Aldo Moro, 53100 Siena, Italy; p.mastroeni@student.unisi.it (P.M.); alfonso.trezza2@unisi.it (A.T.); anna.visibelli2@unisi.it (A.V.); 2 European Reference Network for Hereditary Metabolic Disorders, Department of Biotechnology, Chemistry and Pharmacy, University of Siena, Via Aldo Moro, 53100 Siena, Italy

**Keywords:** alkaptonuria, autophagy, metabolic disease, oxidative stress, lysosomes, homogentisic acid

## Abstract

Alkaptonuria (AKU) is a rare metabolic disorder caused by homogentisate 1,2-dioxygenase deficiency, leading to systemic accumulation of homogentisic acid (HGA) and progressive tissue degeneration characterized by dark urine, ochronosis, and severe osteoarthropathy. Chronic exposure to HGA promotes oxidative stress, chondroptosis, secondary amyloidosis, and impaired autophagy, an essential process for maintaining chondrocyte homeostasis. This study investigated the mechanisms potentially involved in autophagy dysregulation in AKU using the human C20/A4 chondrocyte line treated with 0.1 mM HGA, an established in vitro model of the disease. The findings were then verified using chondrocyte cells and cartilage tissue obtained from AKU biopsies. HGA treatment induced a time-dependent increase in oxidative stress, evidenced by elevated ROS levels, 4-HNE accumulation, and overproduction of mitochondrial superoxide. Autophagy assessment showed an early increase in autophagy-related markers, with increased LC3 and p62 expression and enhanced lysosomal biogenesis (LAMP1). However, prolonged HGA exposure was associated with reduced LC3/LAMP1 colocalization, persistent p62 accumulation, altered acidic compartment staining, and accumulation of autophagy-related structures, supporting a dysregulation of the autophagy–lysosomal pathway. Live-cell imaging further supported a transition from functional autophagy to lysosomal failure under chronic oxidative stress. Overall, this study suggests that prolonged HGA exposure disrupts the interplay between oxidative stress and autophagic flux. The progressive collapse of these adaptive mechanisms may contribute to chondrocyte degeneration and to the pathogenesis of cartilage damage in AKU.

## 1. Introduction

Alkaptonuria (AKU) is an ultra-rare autosomal recessive metabolic disorder caused by mutations in the homogentisate 1,2-dioxygenase (HGD) gene, resulting in a loss of HGD enzymatic function [1]. As a consequence, homogentisic acid (HGA) accumulates systemically and deposits within connective tissues, giving rise to the characteristic dark pigmentation associated with the disease [1]. This pigmentation, called “ochronosis,” primarily involves the osteoarticular system, including cartilage, bone, synovia, and tendons; this condition leads to tissue degeneration, chronic oxidative stress and inflammation, and ochronotic arthropathy [2]. Alkaptonuric cells such as chondrocytes also accumulate intracellular ochronotic pigment [3], sequestered within membrane-bound vesicles in the cytoplasm [4]. Comprehensive analyses have demonstrated that AKU is a multisystemic disorder [1], with ochronotic deposits detected across multiple tissues and implicated in widespread organ dysfunction [1,2]. AKU has further been associated with secondary amyloidosis [5,6] and angiogenesis [7]. Ochronosis is strongly linked to the generation of free radical species, which contribute to oxidative tissue damage and structural degeneration [8,9].

Cartilage is among the most severely affected tissues in AKU [10]. Chondrocytes, the only cell type present in articular cartilage, are responsible for extracellular matrix synthesis and turnover. Because cartilage is a post-mitotic and avascular tissue, chondrocyte proliferation is extremely limited and metabolic turnover is slow [11,12]. In AKU, chondrocytes display structural and functional abnormalities, including aberrant expression of cytoskeletal proteins [4], dysregulation driven by HGA-induced oxidative stress [9,10,13], reduced primary cilia length, and impairment of Hedgehog signaling [14]. In addition, HGA induces chondroptosis, a specific form of apoptosis in AKU chondrocytes, accompanied by distinct morphological alterations [15].

Despite these well-characterized pathological features, the intracellular mechanisms underlying chondrocyte dysfunction in AKU remain incompletely understood. Among the processes potentially involved, autophagy is one of the central processes that preserves articular cartilage homeostasis and maintains chondrocyte functionality [16]. It also contributes to sustaining cellular energy balance during stress conditions, including nutrient deprivation [17]. In this low-nutrient and avascular environment, autophagy plays a crucial role by ensuring the intracellular clearance of unnecessary proteins, pathogens, and damaged organelles. Alterations in autophagic flux can therefore compromise cytoprotective mechanisms in cartilage, contributing to disease onset and progression, as exemplified in osteoarthritis (OA), where a marked reduction in autophagy is a characteristic pathological feature [18]. Proper lysosomal activity depends on intact membranes, efficient acidification, regulated biogenesis, and precise membrane fusion events [19]. Oxidative stress can damage lysosomal membranes, disrupt acidification, and reduce enzymatic activity [20]. When lysosomes fail to fuse with autophagosomes or cannot degrade their contents, autophagic flux becomes impaired. The resulting accumulation of autophagosomes and undegraded cargo places further strain on already compromised cellular systems [16]. Lysosomal inefficiency not only affects autophagy but also leads to intracellular accumulation of oxidized proteins and polymerized pigments, conditions conducive to amyloidogenesis and ochronotic pigment formation and accumulation, both of which are observed in AKU-affected tissues and contribute to the disorganization of the extracellular matrix [21]. The result is progressive loss of cellular integrity, reduced synthetic activity, and eventual cell death [20]. Given the critical role of chondrocytes in maintaining extracellular matrix homeostasis, their decline precipitates cartilage degeneration, joint failure, and the debilitating osteoarthropathy characteristic of AKU [22]. Accordingly, the aim of this study was to further investigate HGA-induced autophagy–lysosomal alterations in the C20/A4 chondrocyte model and in patient-derived AKU chondrocytes. Notably, to the best of our knowledge, this work provides the first assessment of lysosomal biogenesis and lysosome-related alterations in AKU, highlighting their potential contribution to disease-associated impairment of autophagy–lysosomal homeostasis.

## 2. Materials and Methods

### 2.1. Cells Cultures and Treatment

The C20/A4 human chondrocyte cell line (Sigma-Aldrich, St. Louis, MO, USA, SCC041) was grown in Dulbecco’s modified Eagle’s medium (DMEM) supplemented with 1% penicillin/streptomycin (P/S) and 10% fetal bovine serum (FBS) at 37 °C in a humidified environment with 5% CO_2_. HGA (≥98% purity, Sigma-Aldrich) was prepared by dissolving the compound in deionized water to produce a 10 mM stock solution. The solution was then diluted in cell culture medium to a final concentration of 0.1 mM. Culture medium, including HGA-containing treatment medium, was replaced twice weekly.

### 2.2. Primary AKU Chondrocytes Isolation and Culture

Primary human alkaptonuric chondrocytes were obtained, after informed consent, from humeral head cartilage fragments derived from a single AKU patient who had undergone surgery for ochronotic arthropathy. The study received approval from the Local Ethics Committee and was conducted according to the principles of the Declaration of Helsinki (64th, 2013). Chondrocyte isolation was performed immediately after surgery as previously described [23]. Cartilage tissue was minced under aseptic conditions and subjected to sequential enzymatic digestion steps at 37 °C, using 10 mL of digestion medium per gram of wet cartilage tissue for each digestion step. The digestion media contained 1 mg/mL hyaluronidase (30 min), 5 mg/mL pronase (1 h), and 2 mg/mL collagenase (1 h; the cell suspension was then filtered twice using 70 μm nylon meshes, washed, and centrifuged for 10 min at 700× *g*. The pellet was then suspended in DMEM supplemented with 1% P/S and 10% FBS, seeded into T75 flasks, and cultured at 37 °C in a humidified atmosphere with 5% CO_2_. Once isolated, AKU chondrocytes were immediately cultured to prevent de-differentiation.

### 2.3. Immunofluorescence Study

C20/A4 human chondrocytes were cultured on black 24-well glass-bottom plates and treated with 0.1 mM HGA at a density of 1 × 10^4^ cells per well for one week (1 W), or 5 × 10^3^ cells per well for two weeks (2 W). Following incubation, the cells were fixed with 4% PFA, permeabilized with 0.2% Triton X-100 and blocked with Normal Goat Serum (5% NGS). The cells were then incubated overnight at 4 °C with the following primary antibodies: anti-LAMP1 mouse monoclonal antibody (1:50, Invitrogen, Carlsbad, CA, USA), anti-p62/SQSTM1 rabbit polyclonal antibody (1:100, Proteintech, Rosemont, IL, USA), anti-LC3B rabbit polyclonal antibody (1:200, Novus Biologicals, Centennial, CO, USA), and anti-4-HNE mouse monoclonal antibody (1:50, Invitrogen). Afterwards, the cells were incubated for 45 min at room temperature with Alexa Fluor™ 568 goat anti-mouse IgG (1:1000, Thermo Fisher Scientific, Rockford, IL, USA) and Alexa Fluor™ 488 goat anti-rabbit IgG (1:500, Thermo Fisher Scientific). Finally, the samples were stained using DAPI. Images were captured with a fluorescence microscope (Leica DMI8, Wetzlar, Germany), and fluorescence intensity was quantified using ImageJ software (Version 1.54). A quantitative colocalization and correlation study of LAMP1 with LC3B and p62/SQSTM1 was conducted using Icy software (Version 2.5.4) and the GcoPS plug-in [24]. All fluorescence images used for fluorescence intensity and colocalization analyses were acquired under identical microscope settings across experimental groups. For both fluorescence intensity quantification and colocalization analysis, five randomly selected fields were analyzed for each replicate. Signal thresholds were then determined automatically for each channel using Otsu’s method, and the resulting masks were uniformly applied to all images before colocalization quantification.

### 2.4. Live Cell Imaging Study

AKU cartilage fragments and primary chondrocytes were isolated immediately upon surgical collection, and cultured on black 24-well glass-bottom plates. C20/A4 human chondrocytes were cultured on 96-well slides at a density of 5 × 10^3^ cells per well and treated with 0.1 mM HGA for 24 h, on black 24-well glass-bottom plates at a density of 1 × 10^4^ cells per well and treated with 0.1 mM HGA for 1 W, or 5 × 10^3^ cells per well and treated with 0.1 mM HGA for 2 W. Following treatment, live cells were stained with the following dyes for 30 min: MitoSOX Red (500 nM, Invitrogen), LysoTracker Red DND-99 (50 nM, Invitrogen), NucBlue Live ReadyProbes Reagent (2 drops/mL, Invitrogen). Images were captured with a fluorescence microscope (Leica DMI8, Wetzlar, Germany), and fluorescence intensity was quantified using ImageJ software.

### 2.5. Quantification of Intracellular ROS Formation

ROS production in C20/A4 cells was assessed in 96-well plates using 2′,7′-dichlorodihydrofluorescein diacetate (DCFH_2_-DA, Sigma-Aldrich), which is intracellularly deacetylated and oxidized to highly fluorescent 2′,7′-dichlorofluorescein (DCF) [25]. The cells were treated with 0.1 mM HGA for 24 h. DCFH_2_-DA (5 µM), dissolved in Hank’s Balanced Salt Solution (HBSS), was then added to the cells and incubated for 10 min at 37 °C. Fluorescence was measured using a ClarioStarPlus microplate reader (BMG Labtech, Ortenberg, Germany) with excitation at 485 nm and emission at 535 nm. To visualize DCF fluorescence, samples were observed under a fluorescence microscope with a FITC filter (Leica DMI8, Wetzlar, Germany), and images were acquired to confirm the results. The number of cells in each well was determined using a Crystal Violet assay [26]. Results were normalized to the relative cell count for each well and expressed as relative ROS production compared to the untreated group.

### 2.6. Western Blotting

C20/A4 human chondrocytes were treated with 0.1 mM HGA for 1–2 W. Both C20/A4 and AKU primary chondrocytes were then lysed with RIPA buffer supplemented with phosphatase and protease inhibitors and disrupted by sonication for 5 min. Protein concentration was evaluated according to Bradford. A total of 20 µg of cell protein lysate was resolved by 10% sodium dodecyl sulphate-polyacrylamide gel electrophoresis (SDS-PAGE) and then electrotransferred onto a nitrocellulose membrane (0.45 µm pore size; Cytiva). After blocking for 2 h with 5% non-fat dry milk in Tris-Buffered Saline buffer (TBS) at room temperature, membranes were incubated with an anti-LAMP1 rabbit polyclonal antibody (1:500, Invitrogen) and anti-GAPDH HRP-conjugated antibody (1:50,000, Sigma-Aldrich) at 4 °C overnight. The membranes were then incubated with anti-rabbit HRP-conjugated secondary antibody (1:80,000, Sigma-Aldrich) for 1 h at room temperature. Immunoreactive bands were revealed with Luminata Crescendo (Merck Millipore, Burlington, MA, USA) and images were acquired using Image-Quant LAS4000 (GE Healthcare, Milano, Italy). The optical densities of the immunoreactive bands were analyzed using ImageQuantTL software V 7.0 (GE Healthcare). Protein levels were normalized against GAPDH, which served as a protein-loading control.

### 2.7. Statistical Analysis

The results are derived from three independent experiments. Statistical analyses were carried out with GraphPad Prism 10.0 software (GraphPad Software, San Diego, CA, USA). The data are expressed as mean ± standard deviation (SD) and were compared using the unpaired *t*-test or one-way analysis of variance (ANOVA) followed by a post hoc test. A *p*-value of 0.05 or less was considered statistically significant.

## 3. Results

### 3.1. HGA Induces Chronic Oxidative Stress in Chondrocytes

**Figure 1:**
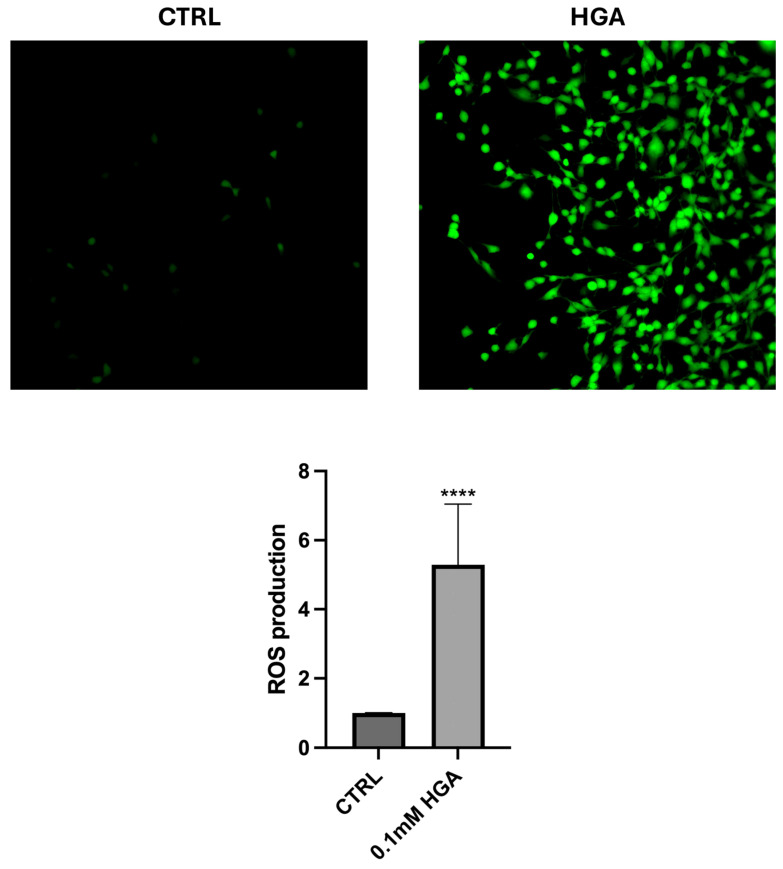
Assessment of ROS production by DCFH_2_-DA probe (green fluorescence) in C20/A4 control and 0.1 mM HGA-treated cells. Data are presented as bar graphs for ROS levels measured from fluorescence intensity normalized to cell count using Crystal Violet assay. Data are displayed as mean ± SD and reported as fold change. An unpaired *t*-test was used to assess statistically significant differences (**** *p* < 0.0001). Cells are shown at 20× magnification.

HGA causes oxidative stress in chondrocytes, ultimately leading to chondroptosis [16]. Its effects have been evaluated under both acute and chronic conditions. C20/A4 human chondrocytes were treated with 0.1 mM HGA, a concentration consistent with levels observed in alkaptonuric patients [27], which effectively replicates disease-related events without compromising cell viability [16]. ROS serve as signaling molecules that play a critical role in the development of stress conditions and are typically produced as part of the early response [28]. In chondrocytes, ROS production is also involved in regular processes such as the regulation of extracellular matrix homeostasis [29]. Therefore, we investigated the impact of HGA on intracellular ROS production. C20/A4 cells were treated with HGA for 24 h. DCF fluorescence exhibited a notable increase in HGA-treated cells compared to the control, indicating the induction of oxidative stress already after 24 h (Figure 1).

**Figure 2:**
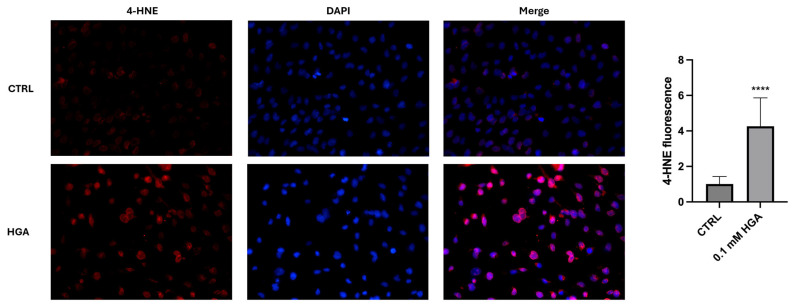
Assessment of 4-HNE expression in control and HGA-treated C20/A4 chondrocytes after 2 W of treatment by immunofluorescence staining. Nuclei were counterstained with DAPI. Fluorescence intensity was normalized to the number of nuclei. Data are displayed as mean ± SD and reported as fold change. An unpaired *t*-test was used to assess statistically significant differences, (**** *p* < 0.0001). Cells are shown at 40× magnification.

Long-term oxidative stress is commonly associated with chronic pathological conditions, leading to DNA and lipid damage, which in turn results in structural alterations and loss of cellular stability and functionality [16]. Among the major markers of lipid peroxidation (LPO), 4-Hydroxynonenal (4-HNE), a specific aldehyde product, has been extensively studied. Notably, 4-HNE accumulation has been shown to be particularly prominent in cartilage from AKU patients, especially in regions exhibiting ochronotic pigmentation [15]. To evaluate LPO, the expression of 4-HNE was investigated by immunofluorescence. C20/A4 cells were treated with HGA for 2 W upon reaching sub-confluence. The expression of 4-HNE was assessed through ImageJ software, normalized to the number of nuclei. HGA-treated cells showed a significant increase in 4-HNE expression compared to the control, revealing LPO-induced damage to cellular lipid structures (Figure 2).

**Figure 3:**
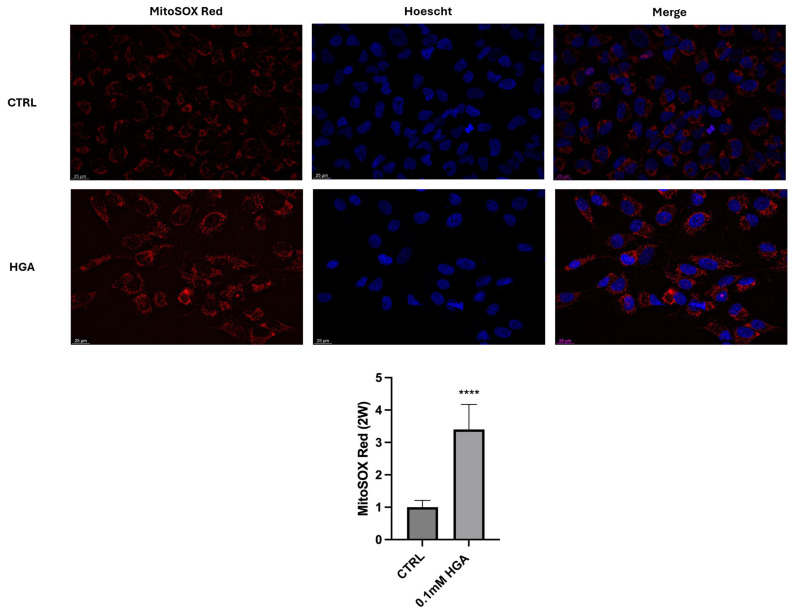
Assessment of mitochondrial superoxide fluorescence in control and HGA-treated C20/A4 chondrocytes after 2 W of treatment by live cell imaging using MitoSOX Red fluorescent dye. Nuclei were counterstained with Hoechst. Fluorescence intensity was normalized to the number of nuclei. Data are displayed as mean ± SD and reported as fold change. An unpaired *t*-test was used to assess statistically significant differences, (**** *p* < 0.0001). Scale bar = 25 μm.

Prolonged oxidative stress is known to cause alterations in the mitochondrial membrane potential, thereby compromising mitochondrial efficiency and contributing to further ROS generation [30]. To assess mitochondrial ROS production, C20/A4 cells were treated with HGA for 2 W before being stained with MitoSOX Red, a mitochondrial superoxide marker. MitoSOX Red fluorescence was assessed using ImageJ software, normalized to the number of nuclei. In HGA-treated cells, MitoSOX Red showed an increase in fluorescence after 2 W of treatment, compared to the control (Figure 3).

### 3.2. HGA-Induced Autophagic Flux Alteration in the C20/A4 Cell Model

**Figure 4:**
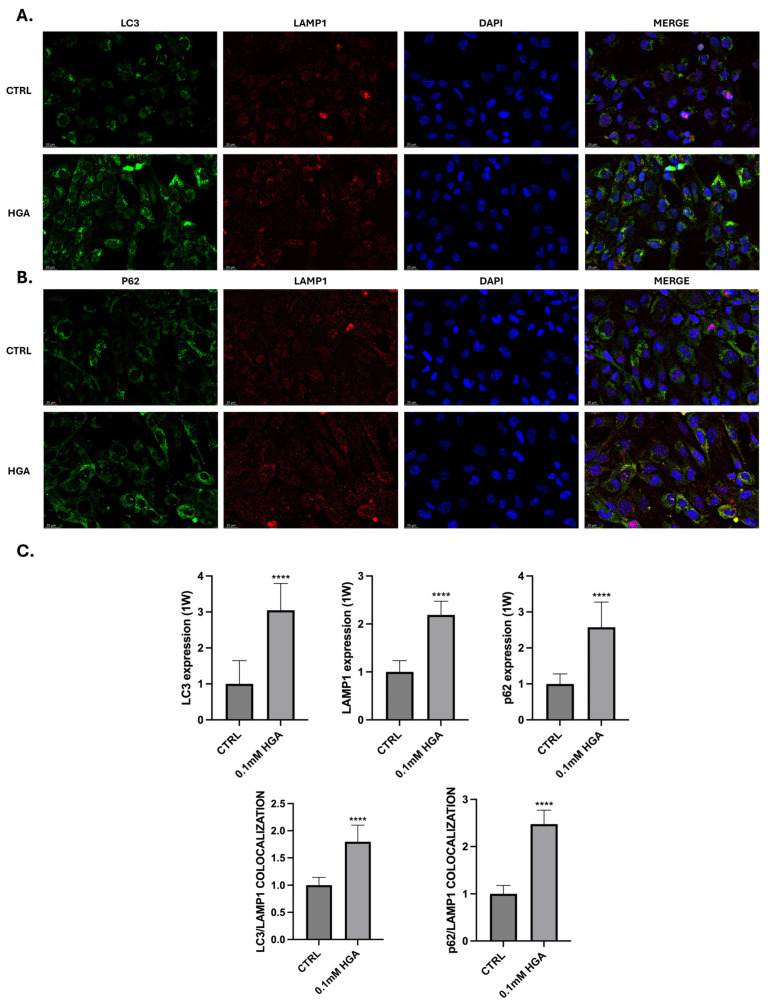
C20/A4 chondrocytes were treated with 0.1 mM HGA for 1 W. (**A**) LC3 (green) and LAMP1 (red) markers are both double-labeled. LC3 and LAMP1 expression, and their colocalization, were increased in HGA treated cells compared to control. (**B**) p62 (green) and LAMP1 (red) are both double-labeled. p62 and LAMP1 expression, and their colocalization increase in HGA treated cells compared to control. HGA-Treated cells showed more specific and puncta signal. Nuclei were counterstained with DAPI. (**C**) Fluorescence intensity was normalized to the number of nuclei. Statistically significant differences are shown using **** *p* < 0.0001. Data are displayed as mean ± SD and reported as fold change. *p*-values were calculated using *t*-test. Scale bar = 25 μm.

LC3, LAMP1, and p62 are key markers of autophagic flux. LC3 transitions from its cytosolic form to the membrane-bound LC3-II, marking autophagic vesicles [17]. LAMP1 is essential for lysosomal integrity and autophagosomes-lysosome fusion [31]. p62 acts as a selective autophagy receptor whose accumulation typically reflects impaired downstream degradation [32]. In this context, it was assessed how prolonged HGA exposure affects these components and the overall progression of the autophagic pathway in C20/A4 cells after 1–2 W of treatment. After 1 W of treatment, HGA caused an increase in LC3 and p62 fluorescences compared with untreated samples (Figure 4). These findings, together with the increase in LAMP1 signal, indicated that HGA acted as an inducer of autophagic flux. To further confirm these data, statistical colocalization analysis was performed using Icy software (GcoPS plugin); colocalization increased both between LC3 and LAMP1 and between p62 and LAMP1, confirming enhanced association between autophagosome, ubiquitinated proteins, and lysosomes, thereby suggesting active autophagy flux.

**Figure 5:**
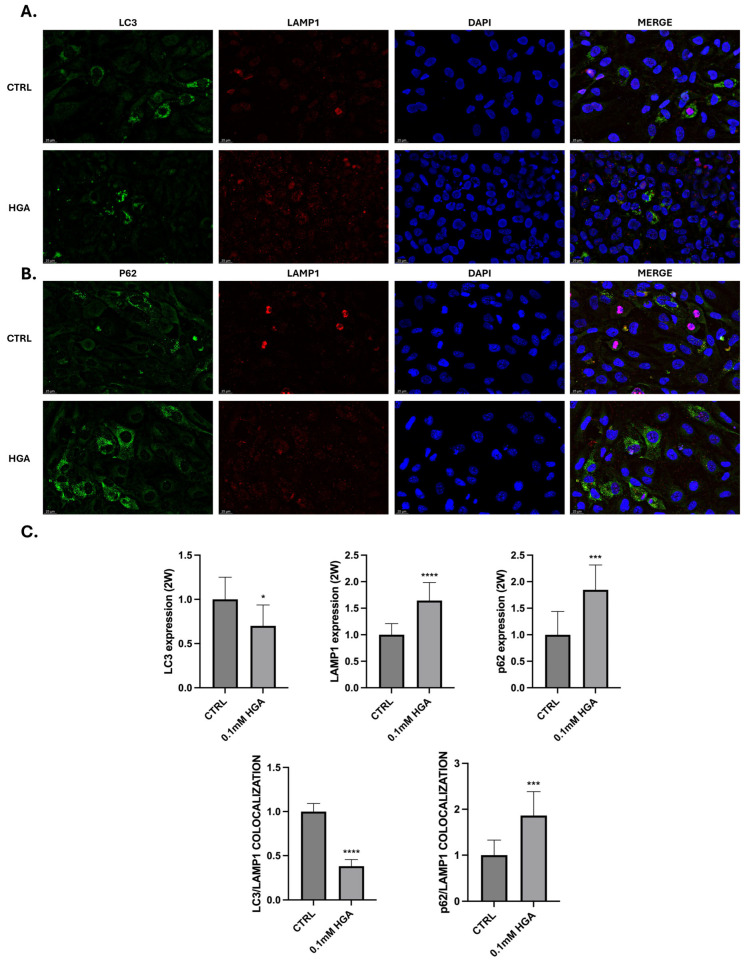
C20/A4 chondrocytes were treated with 0.1 HGA for 2 W. (**A**) LC3 (green) and LAMP1 (red) markers are both double-labeled. LAMP1 expression resulted increased, while decreased the expression of LC3 and its colocalization with LAMP1, in HGA treated cells compared to control. (**B**) p62 (green) and LAMP1 (red) are both double-labeled. p62 and LAMP1 expression, and their colocalization increase in HGA treated cells compared to control. Nuclei were counterstained with DAPI. (**C**) Fluorescence intensity was normalized to the number of nuclei. Statistically significant differences are shown using * *p* = 0.0106, *** *p* < 0.001, **** *p* < 0.0001. Data are displayed as mean ± SD and reported as fold change. *p*-values were calculated using *t*-test. Scale bar = 25 μm.

After confirming the induction of autophagy, the long-term autophagic response of C20/A4 cells to HGA treatment was evaluated. After 2 W of treatment, HGA caused a decrease in LC3 expression compared with the untreated samples, suggesting an impairment of autophagic flux (Figure 5). LAMP1 fluorescence increased in treated cells, indicating enhanced lysosome formation. The colocalization score (GcoPS) revealed that LC3 and LAMP1 showed decreased colocalization, while p62 and LAMP1 maintained a higher colocalization score compared with control cells.

### 3.3. HGA Treatment Increased Lysosome Generation in C20/A4 and AKU Chondrocytes

**Figure 6:**
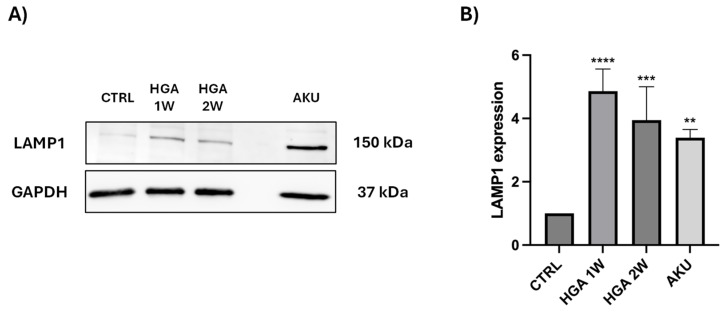
Evaluation of LAMP1 expression in C20/A4 cells treated with 0.1 mM HGA over a two-week period and in AKU chondrocytes. (**A**) LAMP1 expression levels were determined via Western blot analysis. (**B**) Data are reported as fold change. Statistically significant differences are indicated by ** *p* = 0.0029, *** *p* = 0.0002 and **** *p* < 0.0001, using one-way ANOVA followed by Dunnett’s post hoc test.

LAMP1 is a transmembrane glycoprotein primarily located in the lysosomal membrane [33,34]. It plays a key role in maintaining lysosomal integrity, regulating lysosome formation and supporting essential biological processes such as autophagy, endocytosis and cellular homeostasis [33,35]. To investigate lysosome generation in AKU, C20/A4 cells were treated with HGA for 1–2 W. The protein expression of LAMP1 was quantified via Western blot analysis and compared with protein expression observed in chondrocytes obtained from AKU biopsies. Quantitative analysis of immunoreactive bands revealed increased LAMP1 expression compared with untreated C20/A4 chondrocytes (Figure 6).

### 3.4. Chronic HGA Exposure Affects Lysosome-Associated Acidic Compartment Staining

**Figure 7:**
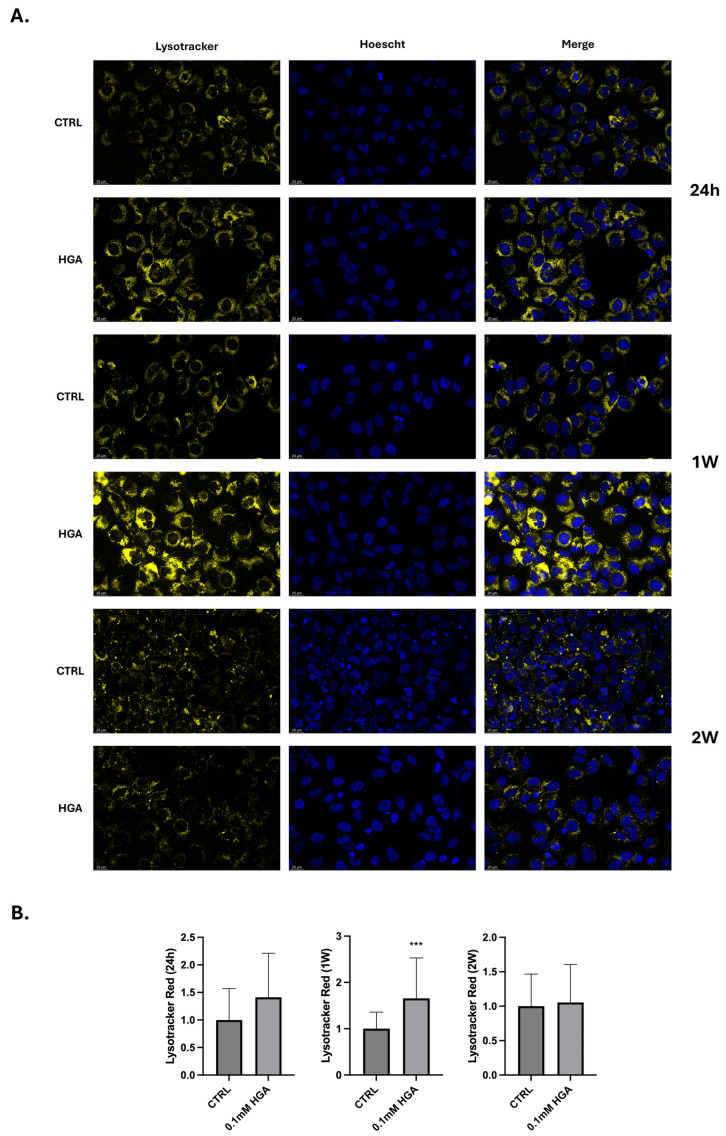
(**A**) Observation and distribution of active lysosomes. (**B**) Quantification of Lysotracker fluorescence showed increased lysosome intensity and distribution after 1 W of HGA treatment, and an inverted trend after long term exposure to HGA, evidencing an alteration to the acid lysosomal compartment. Nuclei were counterstained with Hoechst. Fluorescence intensity was normalized to the number of nuclei. Statistically significant differences are shown using *** *p* < 0.001. Data are displayed as mean ± SD and reported as fold change. *p*-values were calculated using *t*-test. Scale bar = 25 μm.

Lysosomes are intracellular membrane-bound organelles with a key role in degrading and recycling cellular waste, enabled by their hydrolytic enzymes and characteristically low luminal pH (4.5–5) [36], and play a key regulatory role in the maintenance of cell homeostasis [37]. Alterations in lysosomal function can result in impaired clearance of toxic cellular waste, induction of inflammation, apoptotic cell death, and dysregulation of cellular signaling pathways [38]. To evaluate lysosomal activity, and to monitor changes over time, C20/A4 cells were treated with HGA for 24 h, 1 W, and 2 W and subsequently stained with LysoTracker Red. LysoTracker Red staining progressively increased after 24 h of HGA treatment, reaching its maximum intensity at 1 W, indicating enhanced lysosomal activation, consistent with the dye’s capacity to label acidic compartments and increased lysosomal activity (Figure 7A). These observations were further supported by ImageJ analysis, which confirmed increased fluorescence intensity and a more pronounced distribution of active lysosomes (Figure 7B). However, after 2 W of treatment, HGA-treated cells exhibited a marked reduction in LysoTracker staining, comparable to that observed in control cells, suggesting altered acidic compartment staining under prolonged oxidative stress conditions.

**Figure 8:**
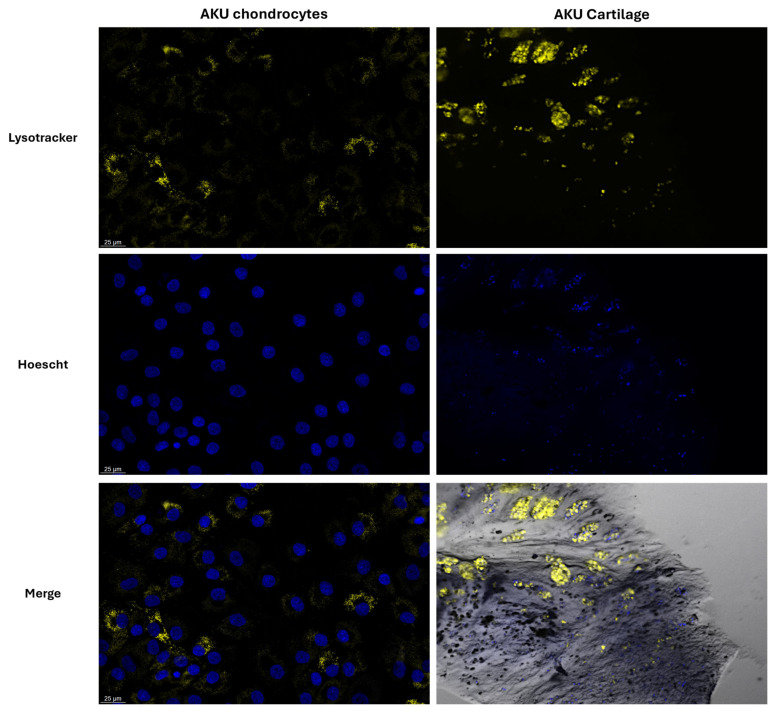
Observation and distribution of active lysosomes in live AKU cartilage and chondrocytes obtained from enzymatic tissue digestion. Lysotracker (yellow) and Hoechst (blue) dyes are both double-labeled. AKU chondrocytes and cartilage tissue showed reduced LysoTracker fluorescence and altered lysosomal distribution compared to the C20/A4 control model. Scale bar = 25 μm.

Acidic intracellular compartment staining was then assessed in primary AKU chondrocytes and living cartilage obtained from a single AKU patient (Figure 8). Consistently with our observations in the C20/A4 cell model after 2 W of HGA treatment, AKU chondrocytes exhibited a reduction in LysoTracker fluorescence and an altered distribution. Cartilage tissue from an AKU patient was cultured and imaged with the same fluorescent dyes. Overall, an alteration in fluorescence intensity and distribution was observed, with areas displaying positive nuclear staining but no detectable Lysotracker signal. This is consistent with altered acidic compartment staining in AKU-affected tissue, in line with chronic HGA-derived oxidative stress.

## 4. Discussion

Alkaptonuria (AKU) is a rare inborn error of metabolism caused by the deficiency of homogentisate 1,2-dioxygenase activity leading to systemic HGA accumulation and a multisystemic debilitating disease whose main features are dark urine, ochronosis, and a severe form of osteoarthropathy [1]. In AKU, chronic oxidative stress induced by HGA progressively impaired autophagy, leading to chondroptosis and ochronotic pigment accumulation [16]. A concomitant HGA-induced alteration of autophagy and accumulation of ochronotic pigment, which recapitulates AKU physiopathological features, has previously been observed [16]. This study aimed to investigate the mechanisms underlying AKU-related autophagy alterations using the human C20/A4 chondrocyte line, which is well established as an in vitro AKU model [39,40]. A concentration of 0.1 mM HGA was selected because it is within the range of HGA levels reported in AKU patients [27] and has previously been shown, under comparable experimental conditions, to induce disease-relevant ochronotic features without compromising cell viability [16].

Oxidative stress in C20/A4 chondrocytes was confirmed by assessing ROS production and 4-HNE expression. Intracellular ROS levels were evaluated by monitoring the oxidation of H_2_DCF-DA after 24 h of HGA treatment. The results showed a marked increase in ROS generation in treated cells compared to control. Long-term oxidative stress was further assessed by immunofluorescence analysis, which revealed increased 4-HNE expression in cells treated with HGA for 2 W, with a characteristic distribution associated with lipidic membranes, confirming chronic oxidative stress condition. Autophagy modulation is crucial for protein and organelle recycling, energy homeostasis, and protection against oxidative stress; moreover, it is heavily affected by ATP availability, especially in chondrocytes, due to the avascular nature of cartilage [41]. Oxidative stress negatively affects mitochondrial function through excessive production of superoxide, a highly reactive species generated as a byproduct of cellular respiration [30]. Excess ROS can damage mitochondrial membranes, proteins, and mitochondrial DNA, thereby impairing the efficiency of the electron transport chain and reducing ATP production [42]. HGA-induced effects on mitochondrial morphology and functionality were investigated by live-cell imaging, through staining with MitoSOX Red Mitochondrial Superoxide Indicator. After 2 W of treatment, MitoSOX Red signal markedly increased, indicating elevated mitochondrial ROS levels, supporting a model in which prolonged HGA exposure induces progressive mitochondrial dysfunction and increased oxidative damage. The data support the hypothesis of an alteration in the autophagic flux. Previous research showed a time-dependent effect of HGA on autophagy regulatory mechanisms in AKU [9]. At first, HGA stimulates autophagy, as a protective response against cellular stress. However, under prolonged exposure, HGA induces oxidative stress and mitochondrial damage, leading to a reduction in autophagic efficiency [22]. As a result, cells are no longer able to maintain homeostasis and progressively undergo chondroptosis [43], accompanied by the accumulation of ochronotic pigment, the hallmark of AKU.

Autophagy alterations were further investigated by immunofluorescence following treatment with HGA for 1 and 2 W. LC3 was selected as an indicator of autophagosome formation [17], p62 as a key adaptor protein involved in the recognition and sequestration of cellular waste [44], and LAMP1 as a marker of the lysosomal membrane, essential for the autophagosome-lysosome fusion [31]. Immunofluorescence analysis showed increased levels of LC3 and p62 in HGA-treated cells after 1 W, confirming the induction of autophagy. An increase in LAMP1 expression was observed after 1 W of treatment, indicating enhanced lysosomal activity, which remained elevated after 2 W. Additionally, after 2 W, LC3 expression was reduced, while p62 accumulated, showing an alteration of the autophagic flux. Colocalization analysis using Icy (GcoPS plugin) and ImageJ (Colocalization plugin) showed that colocalization between p62 and LAMP1 increased in HGA-treated cells, as expected. However, even if colocalization between LC3 and LAMP1 increased after 1 W of HGA treatment, consistent with enhanced autophagic activity, it markedly decreased after 2 W of treatment. This reduction indicates impaired fusion between autophagosomes and lysosomes, potentially due to a reduced generation of lysosomes as suggested by LAMP1 expression, or to defects in autophagosome maturation [45]. Overall, these results are in line with previous investigations from our group performed in the same C20/A4 HGA-treated chondrocyte model, in which LC3 and p62/SQSTM1 alterations were also assessed by Western blot analysis [16]. Together with the present immunofluorescence, colocalization, and LAMP1 Western blot data, these findings support the hypothesis that HGA exposure is associated with autophagy–lysosomal dysregulation in AKU-related chondrocyte alterations.

Western blot analysis revealed striking upregulation of LAMP1 expression in C20/A4 chondrocytes following exposure to HGA (Figure 6). Notably, expression levels peaked after 1 W of treatment, reaching almost five times the level of control cells, suggesting acute activation of lysosomal biogenesis, possibly as a compensatory response to metabolic stress and intracellular accumulation of HGA-derived ochronotic pigment. The persistence of high LAMP1 levels after 2 W of treatment, coupled with the high expression observed in AKU patient-derived chondrocytes, confirms that this is a chronic feature of the AKU cellular phenotype rather than a transient reaction [46,47]. Biologically, LAMP1 overexpression can be interpreted as an attempt by the cell to reinforce lysosomal stability [48]. The substantial glycosylation of the LAMP1 protein serves to shield the lysosomal membrane and acts as a primary marker of lysosomal biogenesis [49]. Since HGA and its oxidation products induce oxidative stress, the cell increases LAMP1 production to prevent lysosomal membrane permeabilization, which would otherwise trigger cathepsin leakage and subsequent cell death [16,46,50]. However, a strong increase in LAMP1, which is usually linked to lysosomal generation and expansion, may also reflect impaired autophagic flux [16]. In this scenario, although the cell produces more lysosomes, as indicated by higher levels of LAMP1, these organelles may be impaired in their function or unable to clear accumulating metabolic waste efficiently [46,47,48].

To further investigate acidic intracellular compartments, time-course analysis of HGA-treated chondrocytes was performed with LysoTracker Red, at 24 h, 1 W and 2 W of treatment. An increase in fluorescence intensity was observed at early time points, with a peak at 1 W. After 2 W of HGA exposure, LysoTracker staining significantly decreased, reaching levels comparable to those of control cells. This was consistent with altered acidic compartment staining under chronic oxidative stress conditions. These findings were further supported by complementary analyses performed in primary chondrocytes and cartilage isolated from a biopsy from an AKU patient. Since these patient-derived analyses were performed using samples from a single AKU patient, they should be interpreted as supportive evidence consistent with the C20/A4 model rather than as an independent quantitative validation. AKU chondrocytes exhibited reduced LysoTracker fluorescence, comparable to that observed in long-term HGA-treated C20/A4 cells, suggesting a similar alteration in acidic compartment staining. To avoid any cell management-related confounding response, living cartilage tissue was cultured directly after surgery, without enzymatic digestion, and then imaged with LysoTracker and nuclear live staining. Areas with positive nuclear staining, but markedly reduced or absent LysoTracker fluorescence were identified, supporting altered acidic compartment staining in AKU-derived tissue. Therefore, further validation in additional AKU samples, together with direct functional assays of lysosomal activity, will be required to better define the mechanistic link between HGA-induced oxidative stress, lysosomal impairment, and autophagy dysregulation.

Overall, the alterations observed in the expression of the autophagy-related markers LC3, LAMP1, and p62, together with changes in their colocalization patterns and the LysoTracker-based staining of acidic compartments, are consistent with dysregulation of autophagy-lysosomal processes in our C20/A4 AKU cell model and in patient-derived AKU chondrocytes.

## 5. Conclusions

This study supports the hypothesis that chronic HGA exposure modifies chondrocyte homeostasis through the induction of oxidative stress and autophagy- and lysosome-related alterations. In the C20/A4 AKU model, HGA induced an early adaptive response characterized by changes in autophagy-related markers and lysosomal biogenesis, followed by alterations in the autophagy–lysosome axis and in the staining pattern of acidic compartments after prolonged exposure. These changes were also detected in patient-derived AKU chondrocytes and in ex vivo AKU cartilage tissue.

Overall, our results suggest that chronic oxidative stress induced by HGA contributes to the dysregulation of autophagy–lysosomal homeostasis in AKU chondrocytes, potentially promoting defective cellular clearance and cartilage degeneration.

## Data Availability

The data supporting the findings of this study are available from the corresponding author upon reasonable request.
